# Selective Laser Melting and Mechanical Properties of Stainless Steels

**DOI:** 10.3390/ma15217575

**Published:** 2022-10-28

**Authors:** Daniel Gatões, Ricardo Alves, Bernardo Alves, Maria Teresa Vieira

**Affiliations:** CEMMPRE—Centre for Mechanical Engineering Materials and Processes, Department of Mechanical Engineering, University of Coimbra, Rua Luís Reis Santos, 3030-788 Coimbra, Portugal

**Keywords:** additive manufacturing, selective laser melting, stainless steel, AISI 316L, AISI 630 (17-4PH), AISI 420, AISI 440C, carbon content

## Abstract

Metal additive manufacturing (AM) has been evolving in response to industrial and social challenges. However, new materials are hindered in these technologies due to the complexity of direct additive manufacturing technologies, particularly selective laser melting (SLM). Stainless steel (SS) 316L, due to its very low carbon content, has been used as a standard powder in SLM, highlighting the role of alloying elements present in steels. However, reliable research on the chemical impact of carbon content in steel alloys has been rarely conducted, despite being the most prevalent element in steel. Considering the temperatures involved in the SLM process, the laser–powder interaction can lead to a significant carbon decrease, whatever the processing atmosphere. In the present study, four stainless steels with increasing carbon content—AISI 316L, 630 (17-4PH), 420 and 440C—were processed under the same SLM parameters. In addition to roughness and surface topography, the relationship with the microstructure (including grain size and orientation), defects and mechanical properties (hardness and tensile strength) were established, highlighting the role of carbon. It was shown that the production by SLM of stainless steels with similar packing densities and different carbon contents does not oblige the changing of processing parameters. Moreover, alterations in material response in stainless steels produced under the same volumetric energy density mainly result from microstructural evolution during the process.

## 1. Introduction

Metal additive manufacturing (MAM), and particularly selective laser melting (SLM), is an effective way of producing steel metal parts [1]. In SLM, a laser is used to melt and fuse metallic powder particles, layer by layer, to build the desired three-dimensional shape (3D object) [2]. SLM was shown to be able to produce low-carbon stainless steel with good quality and reliability [3]. However, some properties in SLM are primarily dependent on the processing step, since a large number of variables affect the quality of final parts, such as powder characteristics, atmosphere, the response of the material to the volumetric energy density (VED) and the rapid heating and cooling cycles [4,5]. As a result, the complex physical and chemical behavior in the melting pool leads to an anisotropic microstructure and the appearance of voids, affecting the final properties [6,7]. The carbon content must be carefully controlled during the manufacturing process, since its presence and quantity can significantly impact the properties of the resulting steel. While the carbon content of SLM-produced stainless steels can be controlled to some degree and generally leads to better mechanical properties, typically associated with less corrosion resistance [8], these steels have a tendency to form cracks and pores during the SLM process [9,10].

Besides porosity, which has a significant impact on mechanical properties, interstitial defects (chemical composition of powder, processing atmosphere, etc.) during SLM and microstructures can also contribute to substantial variations in the properties of the stainless steels. Thus, it is essential to carefully consider the properties of an SLM-produced alloy before selecting it for application [11,12,13].

Stainless steel 316L has been the subject of many studies since the dawn of SLM [14]. The ease of production associated with the low carbon content, no phase transformation (austenitic matrix) in the SLM process, and good mechanical properties make it a standard material in SLM [15,16], and it was used as a standard stainless steel in this study. SS 630 studies have been growing in the last few years due to its excellent mechanical properties [17]. Note that 420 martensitic stainless steel has also been of significant interest in SLM, since its properties are suitable for specific applications, due to its high strength and corrosion resistance [18]. Consequently, many studies have been published concerning the microstructure, mechanical properties, and roughness of these stainless steels, produced by SLM ([19,20,21] −316L, [22,23,24]−630, [25,26,27]−420). SS 440C is also a martensitic stainless steel, but has high carbon content; it is used in applications where high hardness and corrosion resistance are necessary, such as bearings, knives and automotive parts [28]. As far as the authors are aware, this steel has not been subject to any studies regarding SLM technology. Moreover, a detailed study comparing various stainless steels produced with the same parameters in SLM is not available.

In addition to 316L, the standard, in the present study, three stainless steels with similar chromium contents and various levels of carbon content were processed through SLM, using the same atmosphere and set of SLM parameters, as a way to highlight the role of carbon content in AM mechanical properties. A detailed study of the microstructures of these steels compared with the powder was performed. It is worth noting that powder production was also attained in the same atmosphere. The occurrence of defects and their consequences on the mechanical properties can be highlighted by microcomputed tomography (µCT). This non-destructive technique can be useful to study 3D objects pores, voids and impurities distribution, whatever the material [29,30,31].

## 2. Materials and Methods

Four stainless steel powders with increasing carbon content were selected for this study, attained by gas atomization. The 316L and 630 (17-4PH) stainless steels powder were from SLM Solutions GmbH (SLM Solutions Group AG, Lübeck, Germany), and 420 and 440C powders were from Sandvik Osprey Ltd. (Sandvik AB, Sandviken, Sweden). The powders’ chemical compositions are summarized in Table 1. Particle size and particle size distribution (PSD) were evaluated by laser diffraction spectrometry LDS, Malvern Mastersizer 3000 (Malvern Panalytical, Egham, UK). SEM Quanta 400 FEG STEM (FEI Company, Hillsboro, OR, USA) was used for powder shape-factor evaluation. Powder density was measured (5 measurements per steel) by helium pycnometry with Accupyc 1330 (Micrometrics, Norcross, GA, USA).

X-ray diffraction to evaluate types of phases involved a Philips X’Pert diffractometer (Philips, Egham, UK) at 40 kV, Bragg–Brentano geometry (θ–2θ), cobalt anticathode (λ(k*α*1) = 0.178897 nm and λ(k*α*2) = 0.179285 nm) and a current intensity of 35 mA. The X-ray diffraction scans were carried out from 40 to 100° in steps of 0.025°, with an acquisition time of 1 s per step.

**Figure 1:**
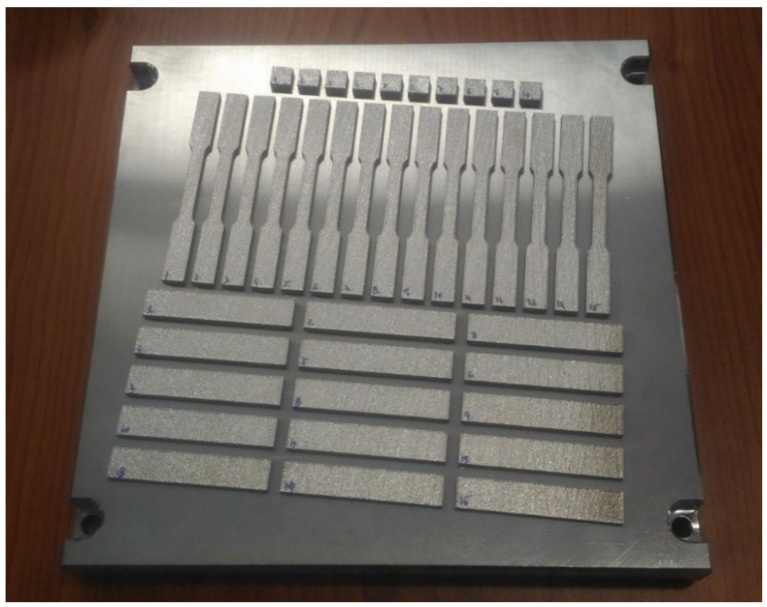
Positions of the specimens on the SLM bed.

The SLM equipment was an EOS M290 system (EOS GmbH, Krailling, Germany) equipped with a Yb-fiber laser (λ = 1064 nm) with a maximum power of 400 W and a spot size of 100 µm. The SLM processing was undertaken with an oxygen content below 0.1% in the working chamber using a continuous flow of nitrogen. The laser power was set to 260 W, scanning speed was 1060 mm/s, hatch space was 100 µm and layer thickness was 30 µm (VED = 82 $\frac{J}{{\mathrm{mm}}^{3}}$). The scanning strategy was a zigzag pattern with a rotation angle of 67° between adjacent layers. Each batch included density cubes (10 × 10 × 10 mm^3^) and tensile test specimens. Additionally, a 10° rotation relative to the substrate position (Figure 1) was added to avoid contamination by spattering. All 3D objects were studied as SLMed, without post-processing treatment.

The final density was evaluated through the Archimedes method and averaged for 10 specimens (density cubes).

Surface and inside defects on tensile specimens were evaluated by X-ray micro-computed tomography using a Bruker SkyScan 1275 (Bruker, Kontich, Belgium). Specimens were polished on both the top and bottom surfaces of tensile test specimens until a thickness of 2 mm was achieved. An acceleration voltage of 100 kV and a beam current of 100 µA were set using a 1 mm copper filter with step-and-shoot mode. Pixel size was set to 10 µm, and the random mode was used. The images were acquired at a 0.4° angular step with 10 frames on average per step using an exposure time of 245 ms. The µCT images were reconstructed with the dedicated manufacturer software.

Optical microscopy was done using a Leica DM 4000 M LED (Leica Microsystems AG, Wetzlar, Germany) with a Leica camera, model MC 120 HD.

For 316L, 630 and 420 steels, etching used a Vilella solution (2 g of picric acid, 5 mL of HCl and 100 mL of ethanol). For 440C, Kalling solution etching (5 g of CuCl_2_, 100 mL of HCl and 100 mL of ethanol) was selected.

Surface roughness was evaluated using focus variation microscopy Alicona Infinite Focus (Bruker, Kontich, Belgium) following ISO 4287 and 4288.

Microhardness measurements were performed on a Fisherscope H100 (Fischer Instrumentation LTD, Pershore, UK), equipped with a Vickers indentor (10 measurements per sample, maximum load of 1000 mN, holding time of 30 s).

**Figure 2:**
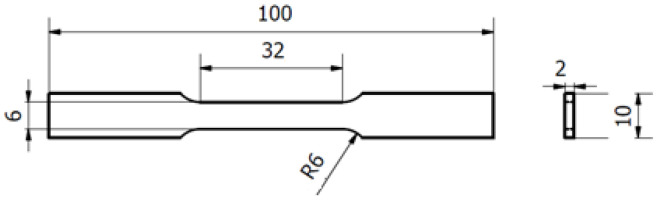
Tensile test specimen measurements, in mm (ISO 6892).

Tensile tests were performed on a SHIMADZU Autograph (Shimadzu, Kyoto, Japan), with a 100 kN load cell, according to ISO 6892, at room temperature, at a strain rate of 10 MPa per second. Tensile specimens’ dimensions were in accordance with Figure 2.

## 3. Results

### 3.1. Powder

All four studied stainless steel powders showed symmetric and narrow normal particle size distributions. Table 2 summarizes the d_10_, d_50_ and d_90_ powder particle size and density.

**Figure 3:**
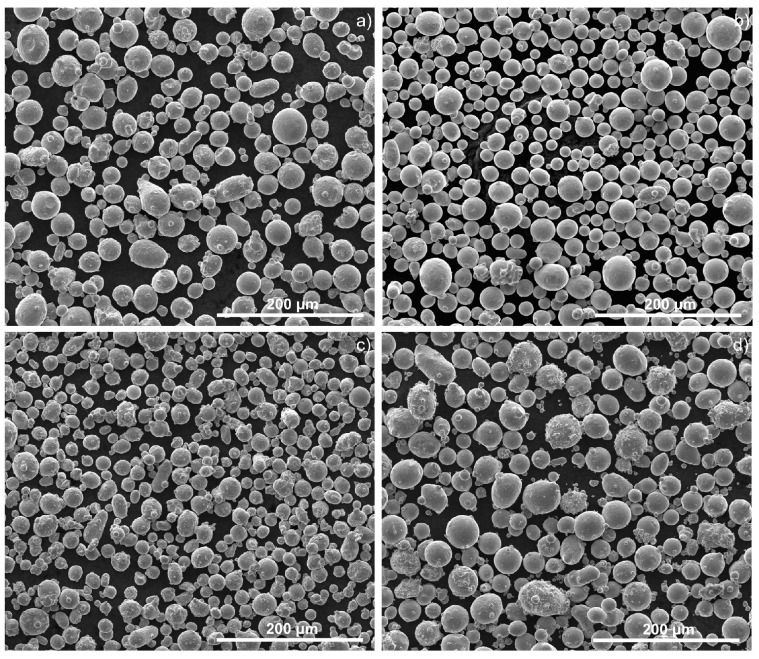
Particle shapes of the 316L (**a**), 630 (**b**), 420 (**c**) and 440C (**d**) powders (SEM).

Powder shape was almost spherical for all powders; there were some satellites, particularly in 316L and 420 steel powders (Figure 3).

**Figure 4:**
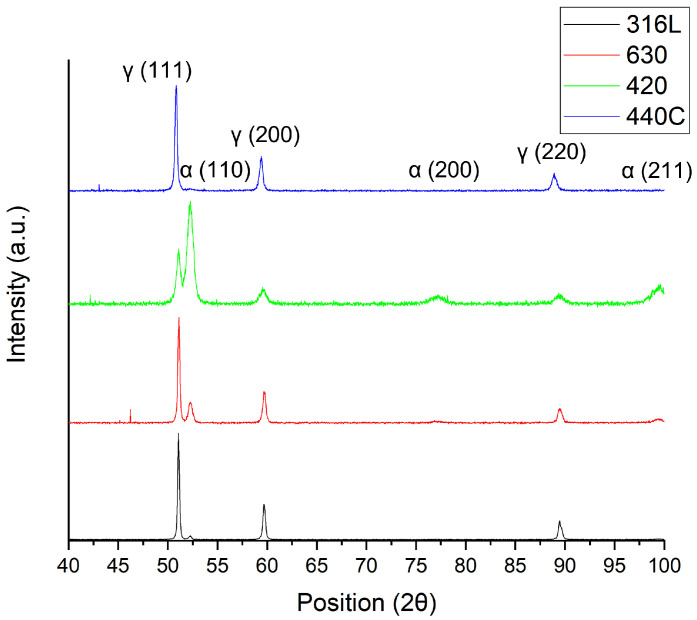
X-ray diffractograms of the 316L, 630, 420 and 440C powders (from bottom to top, respectively).

The selected stainless steels presented martensitic and austenitic phases (Figure 4).

### 3.2. 3D Object

#### 3.2.1. Porosity Evaluation

Porosity is directly related to density. However, the measured values for the four steels are not only related to the porosity, but mainly to the phase difference between the 3D object and starting powder.

**Figure 5:**
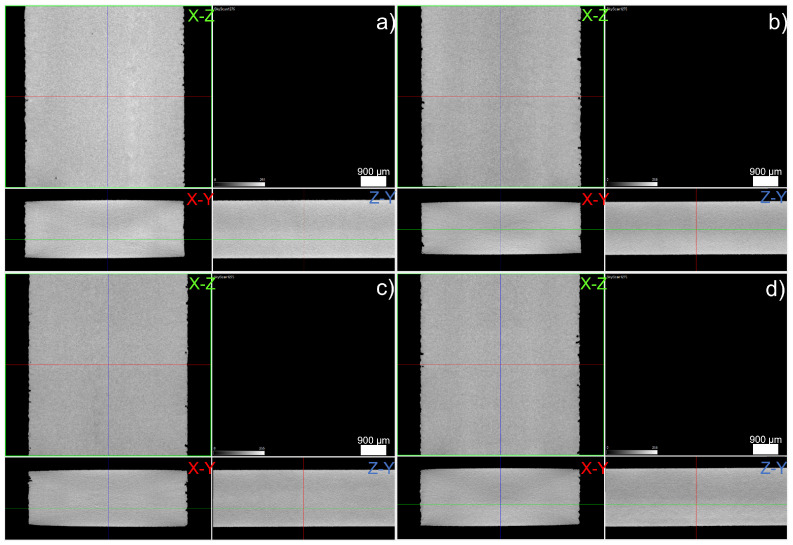
2D visualization of the pores location in cross-section for 316L (**a**), 630 (**b**), 420 (**c**) and 440C (**d**).

**Figure 6:**
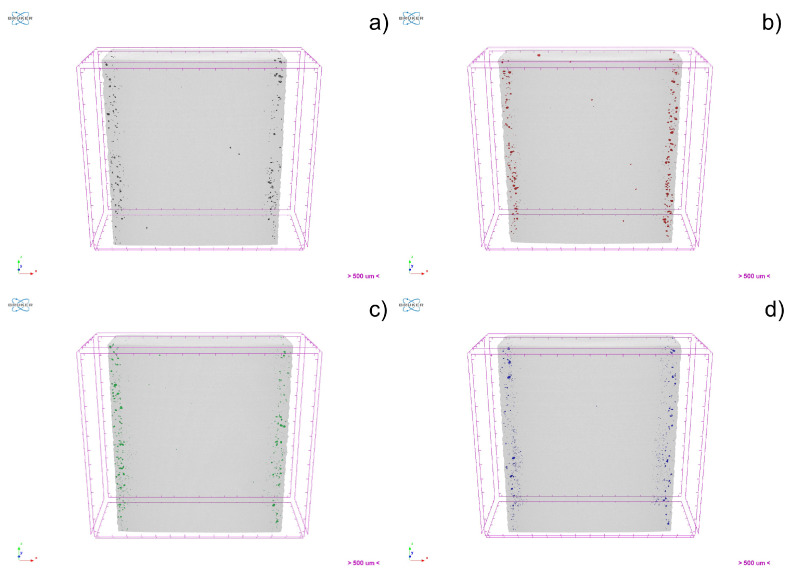
3D visualization of the pores location within the 3D object volume for 316L (**a**), 630 (**b**), 420 (**c**) and 440C (**d**).

The reduced section (cross-section) of the tensile specimen can be observed in Figure 5. Figure 6 shows a rendering of the pore distribution within the reduced section of the tensile specimen. It is noteworthy that only defects bigger than the pixel size (10 µm) were observable in this case.

Table 3 summarizes the values of density of the 3D object and the relative porosity, compared to powder density.

#### 3.2.2. Microstructure

**Figure 7:**
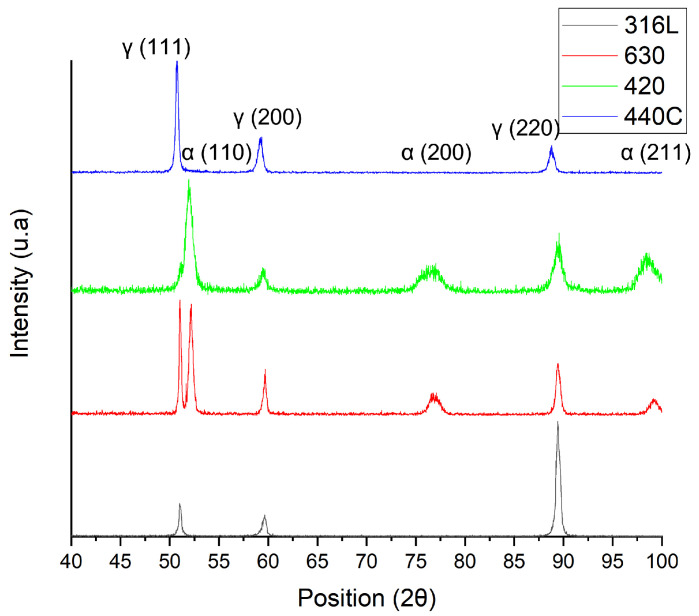
X-ray diffractograms of the 316L, 630, 420 and 440C 3D objects (from bottom to top, respectively).

X-ray diffractions of the 3D objects are shown in Figure 7. Stainless steel 316L showed only an austenitic phase but a strong orientation in [220]. The texture corresponds to <011> direction, which has a major influence on mechanical properties [32]. SS 630 showed an increase in martensite, and 420 showed a shift in the austenite phase when compared to the powder.

**Figure 8:**
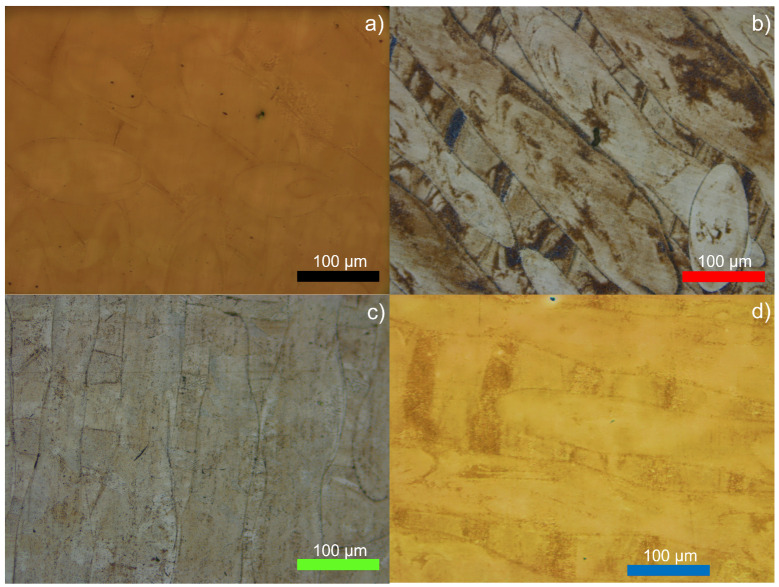
Micrographies of the etched surfaces of 316L (**a**), 630 (**b**), 420 (**c**) and 440C (**d**) stainless steels.

All observed stainless steels, besides SS 316L, tended to form elongated grains in the scanning direction, which is common in metals processed by SLM (Figure 8). It was possible to distinguish narrower elongated grains in 630, 420 and 440C, which allowed the distinction between the previous layer (rotated 90°) and the current layer.

#### 3.2.3. Roughness, Topography and Geometrical Evaluation

Table 4 shows the attained roughness values of each specimen.

**Figure 9:**
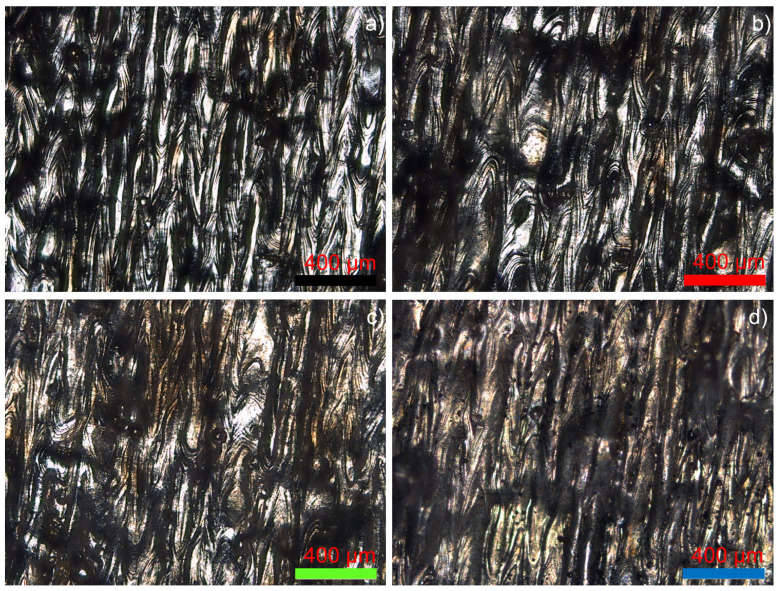
Micrographies of the 316L (**a**), 630 (**b**), 420 (**c**) and 440C (**d**) surfaces.

In what concerns the surface topography, a group of beads corresponding to the scanning direction could be observed in Figure 9. Moreover, the not-fully melted powder could be observed scattered throughout the surface.

Produced tensile specimens were measured in order to ensure the adequacy of mechanical tests. Table 5 summarizes the size comparison between CAD and 3D objects.

#### 3.2.4. Mechanical Properties

#### Microhardness

Table 6 summarizes the measured hardness (HV_0.1_) for the different stainless steels and compares it to the bulk.

#### Tensile Tests

**Figure 10:**
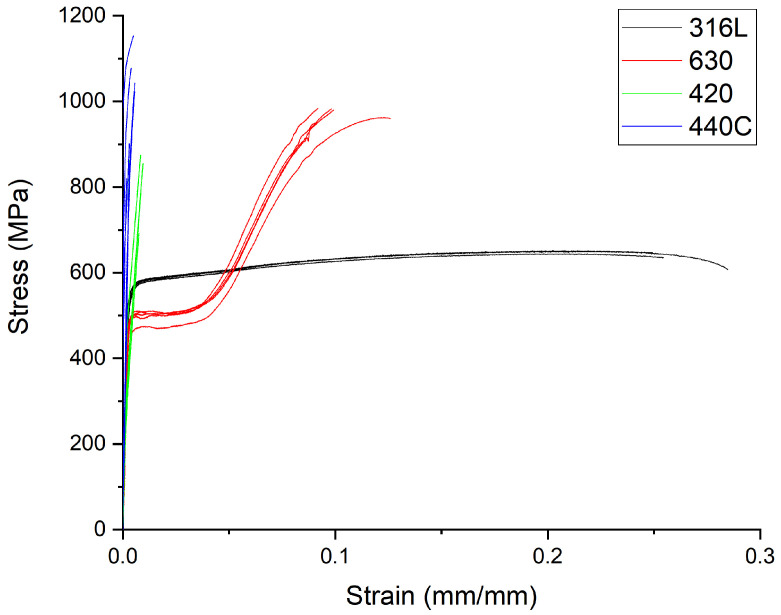
Strain–stress curves for the 316L (black), 630 (red), 420 (green) and 440C (blue) steels.

Figure 10 shows the stress–strain results for five tensile test specimens per stainless steel type.

Table 7 summarizes the elongation and ultimate tensile strength (UTS) of the stainless steel tensile specimens.

## 4. Discussion

### 4.1. Powder

All selected powder particles had similar particle sizes, particle size distributions and shape factors. However, some satellites were observed for 316L and 420 steel powders. These characteristics did not contribute to a significant discrepancy in the powder flowability, and consequently, packing behavior in the powder bed. The difference in measured powder density was mainly related to the phase composition of the steel and its atomization atmosphere. Thus, the austenite phase is always present, resulting from the atomization atmosphere—nitrogen. This is supported by the X-ray diffraction (Figure 4). The SS 440C powder presented mainly the austenite phase and the highest powder density. In opposition, the 420 powder showed the highest martensite content of all selected powder, and therefore, the lowest density.

### 4.2. 3D Object

#### 4.2.1. Density and Microstructure

A representative area of defects (Figure 6) shows that the main pores in all stainless steel 3D objects were relatively similar, independently of carbon content. Surface finishing is mostly obligatory in SLM, since close-to-surface defects can be the main driving force for crack initiation, if not removed. Thus, the presence of these defects is not relevant in real-world applications. However, due to µCT resolution, it can be concluded that microporosity (<10 µm) could also have been present. Nevertheless, this is not enough to justify the measured 3D object’s density.

Considering that the nitrogen atmosphere was similar for powder and 3D objects, and the fact that the carbon content’s effects on the porosity distribution and percentage were negligible, the density must be justified by taking into account the microstructure of the steel (before and after SLM). This means that the density modification can be attributed mainly to the content of the prevalent austenitic phase and the presence of carbide phases, particularly in high-carbon content steels.

SLM processing of the 3D objects, despite 440C not sustaining a phase alteration, affected the phase compositions of 316L, 630 and 420 steels. Particularly, 316L and 420 suffered a shift in the crystallographic orientation, and 630 had an increase in the martensitic phase. This deviation in crystallographic texture resulted from short, deep melt pools, resulting from higher laser power, as shown by [32].

As is common in metals processed by SLM, the microstructure is oriented toward the scanning strategy. This is due to the heating and cooling rates involved in the SLM process, which are the main drivers of microstructure growth. However, high carbon presence in the melt pool was shown to have a particular effect on microstructure due to the carbon movement in the direction of the melt pool frontier, resulting from Marangoni convection [35,36]. Moreover, carbon is a significant element of austenite stabilization. Thus, the carbon depletion of 630 may have been the driver for the increase in martensitic content when compared to the same material powder.

Figure 8 displays apparent grain anisotropy in the 3D objects, whatever the steel. SS 316L’s clear etched surface was consistent with the fact that little-to-no martensite was present in the steel, but grain orientation along the scanning direction was still visible. SS 630 presented a microstructure close to that of dual-phase steel, with austenite and martensite distributed almost equally within the steel and strong processed-caused anisotropy. SS 420 had a dominant martensitic phase with residual austenite. Martensite grew alongside austenite in these steels, as a virtue of the complex melt pool temperature dynamics that occur in the SLM production.

#### 4.2.2. Roughness, Geometry and Topography

Melt pool beads could be seen all over the surfaces. However, it is possible to relate the reduced z-growth of the melt pool with increased carbon content, resulting in lower roughness for higher carbon steels. All steels presented fused powders on their surface, which is detrimental to roughness but can usually be mitigated by shot-peening [37].

The geometrical deviation between the steels, after SLM, was low and consistent, which can mean that the selected VED is indicated for all the steels. VED has a significant effect on melt pool width. If too low, a clear frontier within adjacent melt pools should be observed, along with oriented porosity along the scanning direction. If too high, an uncontrolled melt pool with varying lengths is achieved, and growth in XY direction is expected. Consequently, VED must be considered to be within the optimal range for all the studied stainless steels, corroborating the previous statements.

#### 4.2.3. Mechanical Properties

The microhardness values of the SLM 3D objects produced from the powders of 630, 420 and 440C stainless steels were higher than the maximum hardness of the bulk steels processed by conventional approaches, including heat treatment. Nevertheless, SS 316L showed a slight decrease in hardness compared to the bulk due to the texture assumed during SLM processing. In concomitance with hardness, UTS values for all stainless steels studied, processed by SLM, show a tendency to be higher than the values of conventional processing. It must be highlighted that SS 630 had hardness within the range of the values assumed, considering the various heat treatments that can be used. However, the tensile test showed a particular behavior consistent with the strain-induced martensite formation (TRIP) effect observed by [1,23,24], resulting from a high retained austenite content. This effect led to higher ductility than conventionally heat-treated SS 630. SS 420 and 440C have higher hardness, and the UTS values are outstanding. Nevertheless, SS 420 processed by SLM is a brittle material with negligible elongation due to an unconventional microstructure.

## 5. Conclusions

Whatever the carbon composition, the SLM parameters generally used for 316L induce in other stainless steels with low nickel and higher carbon content low porosity and high densification. The microstructures resulting from SLM without post-processing treatments are mandatory for assessing the mechanical behavior of 3D objects. The hardness and UTS of SLM 3D objects are higher than those of bulk stainless steels with the same compositions, after heat treatment. This is due to a direct relationship between carbon and elements with high affinity to it (e.g., chromium, iron, and molybdenum); however, the content of other elements of selected stainless steels is insufficient for carbide formation. Moreover, it must be highlighted that the stabilization of residual austenite present in the steels with higher carbon content can result from the processing atmosphere; nitrogen is more effective than other elements in the matrix. Furthermore, the strong anisotropy observed in all stainless steels resulted from the selected scanning strategy and VED values. In the studied stainless steels, the microstructural difference, when compared to conventionally processed bulk materials, is mainly due to the processing atmosphere, so a constant VED can be used to process different stainless steels with varying carbon compositions. Future studies regarding the influence of carbon content in indirect additive manufacturing will be compared to the results reported here.

## Figures and Tables

**Table 1:** Chemical compositions of the different stainless steels (wt.%).

Element	C	Cr	Ni	Cu	Mo	Nb + Ta	Si	Mn	N	P	S	O
316L	0.030	16–18	10–14	-	2–3	-	1	2	0.100	0.045	0.030	0.100
630	0.070	15–17	3–5	3–5	-	0.150–0.450	1	1	0.100	-	0.030	0.100
420	min 0.150	12–14	-	-	-	-	1	1	-	0.040	0.030	-
440C	0.950–1.200	16–18	-	-	0.750	-	1	1	-	0.040	0.030	0.100

**Table 2:** Powder size distribution and density of each stainless steel powder.

Powder	d_10_ (µm)	d_50_ (µm)	d_90_ (µm)	$\rho \left(\frac{\mathrm{Kg}}{m^{3}}\right)$
316L	22.7	32.4	45.2	7880
630	17.8	26.2	37.6	7880
420	17.0	24.3	34.3	7820
440C	18.1	26.4	37.9	7940

**Table 3:** Density comparison between powder and 3D object.

Type	Powder Density $\left(\frac{\mathrm{Kg}}{m^{3}}\right)$	3D Object Density $\left(\frac{\mathrm{Kg}}{m^{3}}\right)$
316L	7880	7790
630	7880	7660
420	7820	7590
440C	7940	7490

**Table 4:** Roughness measurements of the tensile test specimens, for each stainless steel.

Type	Ra (µm)	Rq (µm)	Rz (µm)
316L	16.204	19.864	94.401
630	12.988	16.030	79.347
420	7.669	9.440	53.783
440C	6.369	8.093	46.391

**Table 5:** Comparison between CAD and final 3D object sizes of tensile test specimens, for each material type.

Type	Width at Grip (%)	Width at Reduced Section (%)	Length (%)
316L	99.40 ± 0.24	98.50 ± 0.32	99.70 ± 0.03
630	99.60 ± 0.19	99.00 ± 0.27	99.81 ± 0.05
420	99.70 ± 0.25	98.67 ± 0.27	100.00 ± 0.06
440C	99.20 ± 0.03	98.83 ± 0.25	99.58 ± 0.05

**Table 6:** Microhardness levels of the selected stainless steel 3D objects, compared to the bulk [33].

Type	3D Object HV_0.1_	Bulk HV_0.1_
316L	133 ± 17	170–220
630	306 ± 11	250–460
420	647 ± 27	260–641
440C	803 ± 26	510–760

**Table 7:** Elongation and ultimate tensile strength for each steel-type 3D object, compared to bulk [34].

Type	3D Object ϵ (%)	Bulk ϵ (%)	3D Object UTS (MPa)	Bulk UTS (MPa)
316L	25.4 ± 2.4	30–50	645 ± 10	550
630	10.0 ± 1.3	4–6	1020 ± 33	943
420	0.7 ± 0.2	5–11	814 ± 74	655
440C	0.4 ± 0.1	0.5–4	1164 ± 13	760

## Data Availability

Data sharing not applicable to this article.
